# Comparison of distortion correction preprocessing pipelines for DTI in the upper limb

**DOI:** 10.1002/mrm.29881

**Published:** 2023-10-13

**Authors:** Ryckie G. Wade, Winnie Tam, Antonia Perumal, Sophanit Pepple, Timothy T. Griffiths, Robert Flather, Hamied A. Haroon, David Shelley, Sven Plein, Grainne Bourke, Irvin Teh

**Affiliations:** ^1^ Leeds Institute for Medical Research, University of Leeds Leeds UK; ^2^ Department of Plastic, Reconstructive and Hand Surgery Leeds Teaching Hospitals Trust Leeds UK; ^3^ Division of Psychology, Communication & Human Neuroscience The University of Manchester Manchester UK; ^4^ Leeds Teaching Hospitals Trust Leeds UK; ^5^ Leeds Institute of Cardiovascular and Metabolic Medicine, University of Leeds Leeds UK

**Keywords:** anisotropy, diffusion, diffusivity, distortion, DTI, magnetic resonance, median, nerve, pre‐processing, radial, ulnar

## Abstract

**Purpose:**

DTI characterizes tissue microstructure and provides proxy measures of nerve health. Echo‐planar imaging is a popular method of acquiring DTI but is susceptible to various artifacts (e.g., susceptibility, motion, and eddy currents), which may be ameliorated via preprocessing. There are many pipelines available but limited data comparing their performance, which provides the rationale for this study.

**Methods:**

DTI was acquired from the upper limb of heathy volunteers at 3T in blip‐up and blip‐down directions. Data were independently corrected using (i) FSL's TOPUP & eddy, (ii) FSL's TOPUP, (iii) DSI Studio, and (iv) TORTOISE. DTI metrics were extracted from the median, radial, and ulnar nerves and compared (between pipelines) using mixed‐effects linear regression. The geometric similarity of corrected *b* = 0 images and the slice matched T1‐weighted (T1w) images were computed using the Sörenson‐Dice coefficient.

**Results:**

Without preprocessing, the similarity coefficient of the blip‐up and blip‐down datasets to the T1w was 0·80 and 0·79, respectively. Preprocessing improved the geometric similarity by 1% with no difference between pipelines. Compared to TOPUP & eddy, DSI Studio and TORTOISE generated 2% and 6% lower estimates of fractional anisotropy, and 6% and 13% higher estimates of radial diffusivity, respectively. Estimates of anisotropy from TOPUP & eddy versus TOPUP were not different but TOPUP reduced radial diffusivity by 3%. The agreement of DTI metrics between pipelines was poor.

**Conclusions:**

Preprocessing DTI from the upper limb improves geometric similarity but the choice of the pipeline introduces clinically important variability in diffusion parameter estimates from peripheral nerves.

## 1 INTRODUCTION

Peripheral neuropathy and nerve injury are common, affecting 1 in 10 adults over the age of 40.^1^ Diffusion‐weighted MRI (dMRI) characterizes tissue microstructure and provides reproducible^2^, ^3^, ^4^, ^5^ proxy measures of nerve health which are sensitive to axon type, diameter, density, myelination, and organization.^6^, ^7^, ^8^, ^9^ DTI is the most prevalent form of dMRI and this is typically acquired by single‐shot echo‐planar imaging (EPI). However, EPI is susceptible to geometric and intensity distortions due to a combination of susceptibility‐induced field inhomogeneities, eddy currents, subject motion, and low bandwidth in the phase‐encoding direction.

Preprocessing is a multi‐step process concerned with the correction of geometric and signal distortions which are prevalent in EPI data.^10^ The aim is to improve geometric fidelity and minimize false negatives, without increasing false positives in the postprocessing (analysis) phase. Although it is widely accepted that preprocessing should be performed because it improves the accuracy of dMRI metrics and tractography, there are no accepted standards. Consequently, practices and pipelines vary, which adversely affects the alignment of diffusion‐weighted and anatomical images,^11^ generating important differences in tensor based metrics^12^ and tractograms.^2^ Collectively, this negatively impacts the reproducibility of dMRI studies.^13^, ^14^

The majority of the dMRI community agree that preprocessing pipelines should be standardized.^15^ Furthermore, over 80% believe that corrections for artifacts arising from subject motion, eddy currents, field inhomogeneities, and thereafter, *b*‐matrix rotation are required.^15^ Several studies have shown that phase‐encoding‐based methods yield the best corrections of the above artifacts.^14^, ^16^, ^17^ Ideally, full dMRI datasets are acquired in opposing phase‐encoding (PE) directions (that is, all images in both blip‐up and blip‐down directions)^12^ although similar results can be achieved by acquiring non‐diffusion‐weighted (DW) data with reversed PE or via deep‐learning.^18^ There are several open‐source software packages that perform corrections for distortions arising from some or all of the following: motion, eddy currents, and susceptibility‐induced artifacts, using data with opposing phase encoding, such as animaDistortionCorrection,^19^ animaBMDistortionCorrection,^20^ DR‐BUDDI from the TORTOISE suite,^21^, ^22^, ^23^ DSI Studio,^24^ EPIC,^25^ HySCO,^26^ and TOPUP^27^ and eddy^28^ from the FMRIB Software Library (FSL). However, there is uncertainty about which pipeline offers the best performance and most packages were developed for brain imaging, so there is limited work on non‐brain real‐world data.^29^

In this study, we compared the performance of the three most common software packages that offer phase‐encoding‐based preprocesssing of DTI, namely FSL's suite (which is also embedded into the most popular postprocessing package worldwide, MRtrix3^30^), DSI Studio, and TORTOISE.

## 2 METHODS

This cross‐sectional study was designed and reported in accordance with the STROBE and STARD guidance, taking into account the domains of the QUADAS‐2^31^ and PRISMA‐DTA^32^ tools. Approval was provided by the National Health Research Authority (ID 19/NW/0324), and written informed consent was obtained from all participants.

### 2.1 Recruitment

We included 13 healthy participants (8 males and 5 females) of mean age 29 y (SD 6·22, range 21–40). Their mean height was 174 cm (SD 9) and weight 75 kg (SD 17), giving a mean body mass index of 25 (SD 5). Volunteers were recruited opportunistically between July 2019 and March 2020. We excluded volunteers who had non‐MR safe implants, peripheral neuropathy, metallic implants near the elbow or claustrophobia.

### 2.2 Image acquisition

DTI data were acquired at a field strength of 3T using a MAGNETOM Prisma (Siemens Healthcare, Erlangen, Germany) and a single‐shot EPI sequence. Participants were scanned prone, with the shoulder flexed and elbow extended. The elbow was positioned as close to the isocenter of the magnet as comfortably possible. A four‐channel flexible coil was wrapped around the elbow and secured with strapping. Fifty‐five contiguous axial slices of 3 mm thickness were acquired, at an in‐plane resolution of 1.5 mm^2^.^33^ The FOV was reduced to 192 × 78 mm (frequency × phase) using ZOOMit (TimTX TrueShape) with TrueForm B1 shim. We applied 30 non‐collinear monopolar diffusion encoding gradients using a Jones scheme^34^ with the following parameters: *b*‐value 800 s/mm^2^ (*Δ* = 19.0 ms, *δ* = 13.2 ms), four interleaved non‐DW (*b* = 0) images, TE 74 ms, TR 7800 ms, effective echo spacing 0·97 ms and EPI (“turbo”) factor 52 (giving an echo train length of ˜50 ms), GRAPPA off, 6/8 partial Fourier, receiver bandwidth 1184 Hz, distortion correction off and strong fat saturation. Four signals/averages (totaling 120 DWIs and 16 interleaved *b* = 0 images) were acquired which required 17 min 50 s. The sequence was repeated with the (right–left) PE direction reversed. This was supplemented by slice‐matched T1‐weighted (T1w) images.

### 2.3 Preprocessing

**FIGURE 1:**
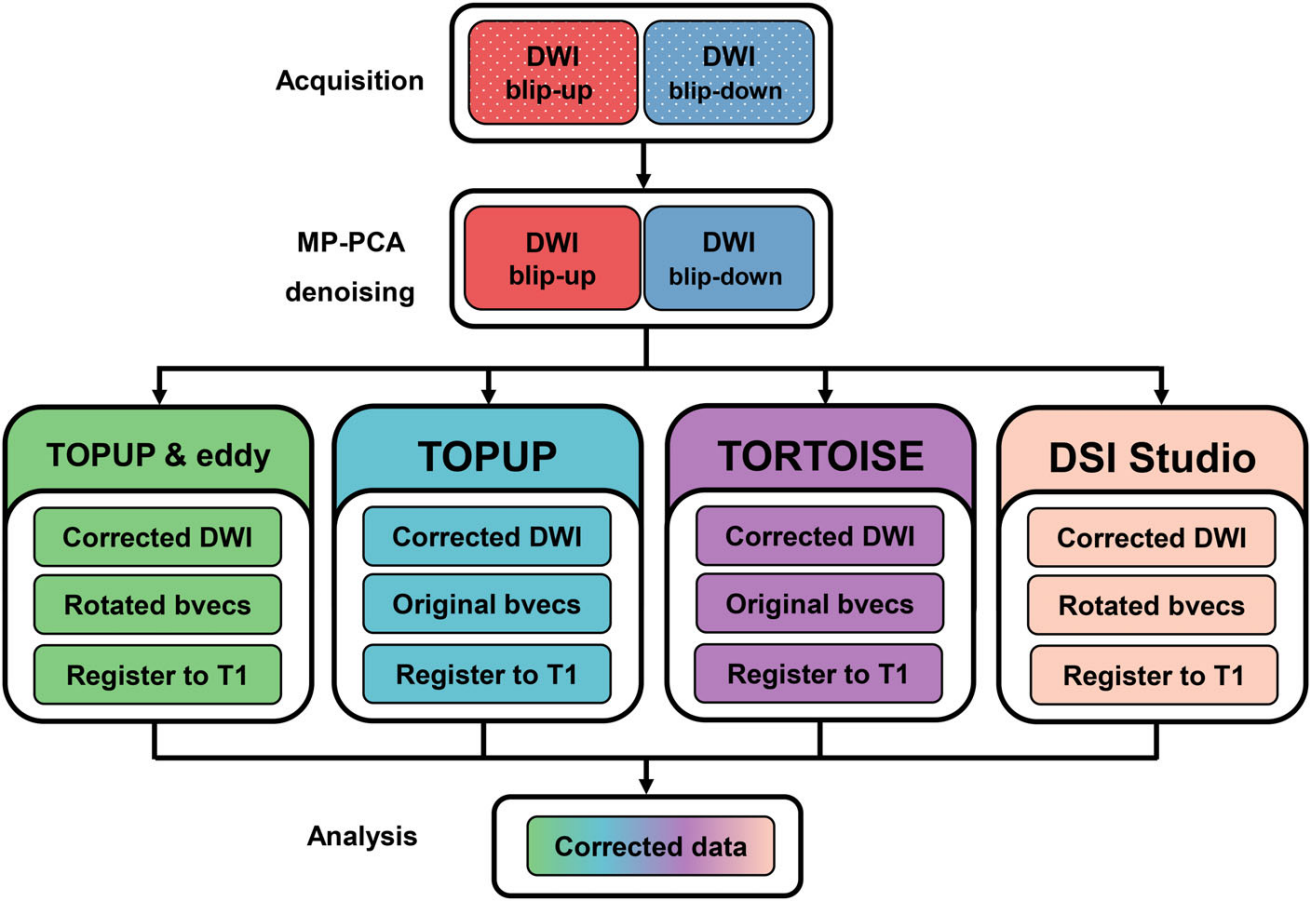
Steps for comparing preprocessing pipelines. Note that both TOPUP & eddy are part of the FMRIB Software Library (FSL). By default, both FSL's eddy and DSI Studio automatically rotated the *b*‐matrix^37^ whereas the analogous function in TORTOISE did not. TOPUP alone did not alter the *b*‐matrix. When registering the dMRI data to the space of T1, DSI Studio automatically further transformed to the *b*‐matrix and saved the newly rotated *b*‐matrix within the native .fib file for analysis.

DICOMs were converted to nifti using dcm2niix^35^ and denoised by MP‐PCA.^36^ Thereafter, data were passed to each pipeline for concatenation and correction (Figure 1).

In DSI Studio^24^ (using the November 16th 2021 release), we used the “Correct AP‐PA scans” option whereby the correction is based on the computed cumulated spin density along the phase encoding direction and applied using point‐to‐point mapping to restore the unwrapped distribution. Next, we applied the “motion correction” option which applies a linear registration based on mutual information between the *b* = 0 images and DWIs. The summative transformation is then applied to the *b*‐matrix. The corrected data were saved in the software specific.fib format (which includes the corrected *b*‐matrix within) and also exported in nifti format.

In FSL v6.0.2, the *b* = 0 images were extracted and passed to TOPUP, using the default configuration file (b02b0.cnf). To test the performance of susceptibility correction only within FSL, the applyTOPUP command was used, supplying the blip‐up and blip‐down data and default parameters.

Again, in FSL v6.0.2, after running TOPUP with the default configuration file (b02b0.cnf), a binary mask (removing only noise) was generated from the corrected mean *b* = 0 images, and these outputs were passed to eddy (eddy_cuda) with the following options and configurations. The eddy current‐induced fields were modeled using a quadratic function (‐‐flm = quadratic). Data of opposing phase were concatenated by least‐squares resampling (‐‐resamp = lsr), which is the default. Outlier slices (with signal intensities at least four SDs from the expected) were replaced with predictions made by Gaussian Processes using the ‐‐repol option^38^; according to the outlier reports, a median 1 slice per volunteer (0.3% of the total slices per volunteer, range 0–11 slices) was replaced. We also enabled the slice‐to‐volume motion correction using the ‐‐mporder option^39^ with 15 degrees of freedom and ‐‐estimate move_by_susceptibility^40^ whereby susceptibility‐induced field change due to subject motion is estimated and corrected using a first‐order Taylor expansion of the static field, with respect to pitch and roll.

Within TORTOISE, DIFFPREP was applied, and data were combined and corrected using Diffeomorphic Registration for Blip‐Up blip‐Down Diffusion Imaging (DR_BUDDI)^22^ using the default settings. The mean *b* = 0 images images were selected as the reference (T2w) dataset. In two volunteers, some slices at the ends of the stack (in the proximal arm and distal forearm regions) could not be corrected, so these specific slices were excluded from postprocessing. We could not identify a rotated b‐table or transformation matrix in the ouput folder of DR_BUDDI, so the original *b*‐vectors were used.

### 2.4 Postprocessing

The corrected datasets from each pipeline were imported to DSI Studio. Datasets were automatically registered to the T1w data using a rigid body transformation and the same transformation was automatically applied to the *b*‐matrix. Diffusion was quantified using restricted diffusion imaging^41^ and reconstructed using Generalized Q‐Space Imaging (GQI).^42^ This model‐free approach was selected to compare the four preprocessing approaches because peripheral nerves are more conspicuous on the resultant quantitative anisotropy (QA) maps, as compared to traditional tensor‐based maps. Also, GQI is readily applicable to numerous different diffusion sampling schemes, the outputs are comparable to more complex q‐space methods and it generates a spin‐density function that is the closest to reality.^42^

### 2.5 Segmentation of the limb

Given the variability of coil position and therefore proximal/distal coverage, we classified a 5.7 cm section (19 axial slices) centered on the radiohumeral joint as the “Elbow” region. Measurements proximal to this were classified as within the “Arm” and measurements distal were classified as the “Forearm”.

### 2.6 Measuring the similarity of the T1w and corrected DTI images

**FIGURE 2:**
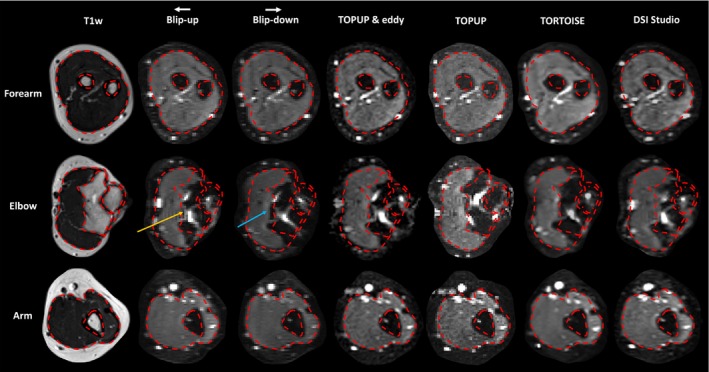
A montage showing slice matched T1‐weighted images, *b* = 0 images from the blip‐up and blip‐down dMRI acquisitions, and corrected *b* = 0 images from each pipeline, in the right limb of a 30‐y‐old female. The phase‐encoding direction (white arrow) and consequential areas of signal compression/pile‐up (gold arrow) and dilation (blue arrow) are shown. The red dotted lines were drawn on the T1w image and overlaid on the *b* = 0 images.

**FIGURE 3:**
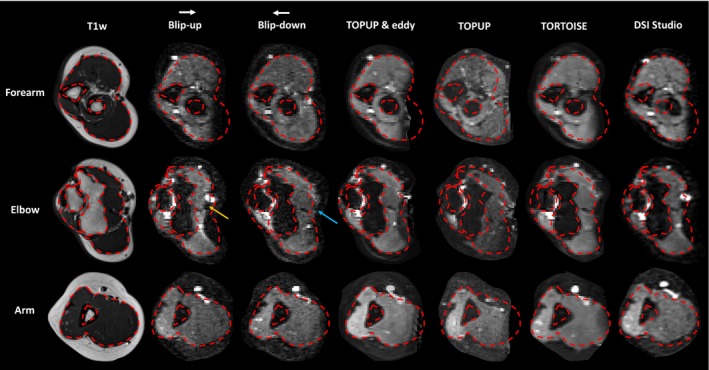
A montage showing slice matched T1‐weighted images, *b* = 0 images from the blip‐up and blip‐down dMRI acquisitions, and corrected *b* = 0 images from each pipeline, in the left limb of a 39‐y‐old female. The phase‐encoding direction (white arrow) and consequential areas of signal compression/pile‐up (gold arrow) and dilation (blue arrow) are shown. The red dotted lines were drawn on the T1w image and overlaid on the *b* = 0 images.

To quantify the morphological similarity of the corrected DTI datasets to the anatomical (T1w) reference image, regions of interest (ROIs) were drawn to enclose the subfascial structures of the upper limb (Figures 2 and 3). The regions were drawn manually on the *b* = 0 images of the raw blip‐up, blip‐down, and corrected dMRI datasets, as well as the slice‐matched T1w images, by RGW. This was performed on matching slices in the arm, elbow, and forearm. The volumetric overlap of these regions were binarized and then used to calculate the Sörenson‐Dice similarity coefficient.^43^, ^44^

### 2.7 Extraction of metrics

To extract metrics from the median, radial, and ulnar nerves within each of the corrected datasets, ROIs were drawn over the center of the ulnar, median, and radial nerves on every QA map by three researchers (WT, AP, and SP; Figure S1). All ROIs were checked independently by RGW, TG and RF. Around the elbow, the normal cross‐sectional area of the ulnar nerve is ∼7mm^2^,^45^, ^46^ the median ∼7 mm^2^, and the radial nerve is ∼5.1mm^2^.^46^ To minimize partial volume effects, the ROI was limited to 1.5 mm^2^ (one voxel) and centered over the cross‐section of the nerve, which typically had the highest regional QA value. The following metrics were then extracted from each ROI throughout the length of the nerve: fractional anisotropy (FA), radial diffusivity (RD), axial diffusivity (AD), and mean diffusivity (MD) and their corresponding maps are shown in Figure S2.

### 2.8 Analysis

The raw data are available open source at https://osf.io/z29m6/ (last accessed September 7, 2023). Data were analyzed using Stata v16/MP (StataCorp LLC, Texas). Normality was confirmed by visualization of the distribution of data, so scaled variables are represented by the mean (and SD). The Sörenson‐Dice coefficients were skewed in one subgroup, so are represented globally by the geometric mean and 95% confidence interval (CI). The effect of different preprocessing pipelines on the Sörenson‐Dice coefficient was explored using mixed‐effects linear regression, given that the residuals were normally distributed. The dependent variables in four separate models were the DTI metrics (FA, MD, RD, and AD). The fixed effect in each model was the method of processing (blip‐up, blip‐down, TOPUP & eddy, TOPUP, DSI Studio, or TORTOISE). Restricted maximum likelihood was used to estimate the cluster‐level (volunteer) variance. The variance and covariance parameters were unstructured and, so, distinctly estimated. The outputs of these models are shown as cubic splines, formatted using grstyle.^47^, ^48^ To summarize agreement between pipelines, the intraclass correlation and Pearson's r coefficients were computed and shown alongside Raincloud^49^ and Bland‐Altman plots. In line with calls for the abolition of *p*‐values, we have minimized their use and avoided the term “statistical significance”,^50^, ^51^ instead focussing on how the findings might translate to future real‐world imaging scenarios.

## 3 RESULTS

**FIGURE 4:**
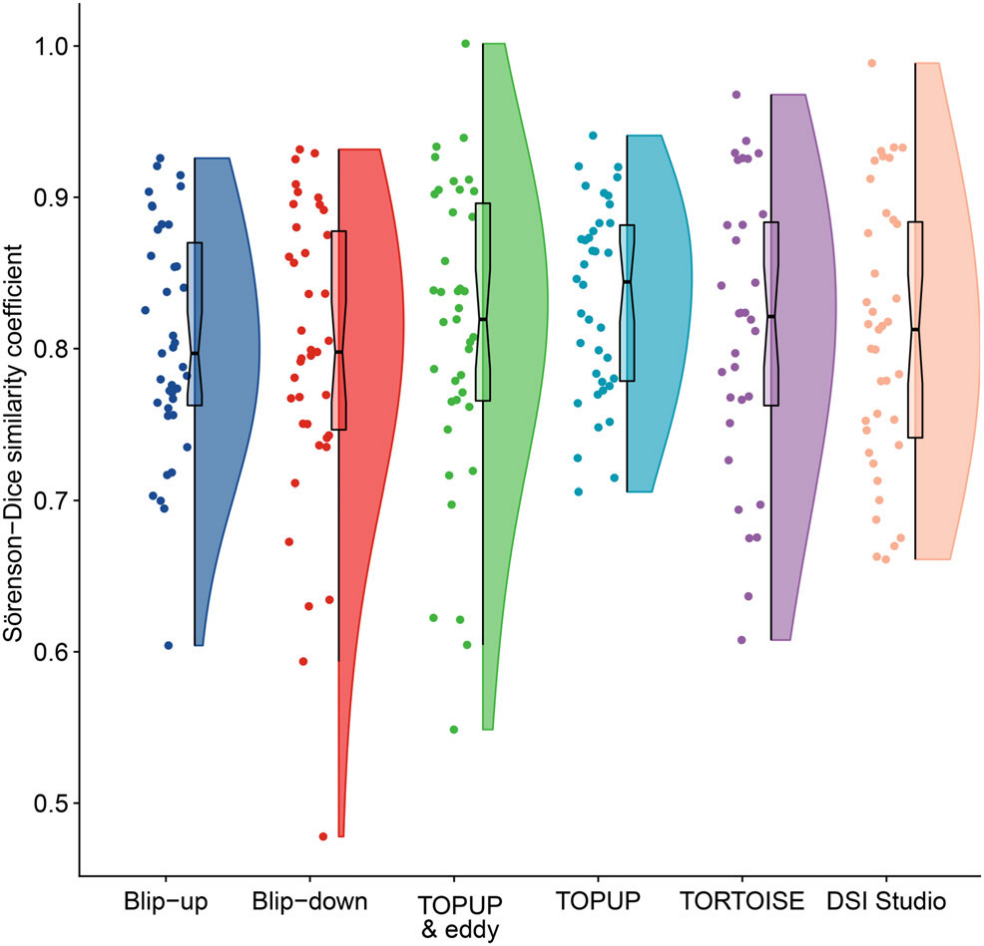
Raincloud plot showing the Sörenson‐Dice similarity coefficient of the raw blip‐up and blip‐down and preprocessed datasets against the T1w reference images.

Without preprocessing, the geometric mean Sörenson‐Dice coefficient of the blip‐up and blip‐down datasets to the T1w was 0.80 (CI 0.78–0.83) and 0·79 (CI 0.75–0.83), respectively. Preprocessing improved the similarity by a mean of 1% (mean Sörenson‐Dice of TOPUP & eddy 0.81 [CI 0.77–0.83], TOPUP alone 0.83 [CI 0.81–0.86], DSI Studio 0·80 [CI 0.77–0.83], and TORTOISE 0.81 [CI 0.78–0.85]; Figure 4) with no meaningful differences between pipelines.

Based on the Sörenson‐Dice coefficients, distortions were worst in the elbow region (0.74 [CI 0.71–0.77]) but similar in the arm (0.82 [CI 0.79–86]) and forearm (0·83 [CI 0.76–0.90], Figure S3). Overall, distortions were better corrected in the arm and forearm (Sörenson‐Dice coefficients were 8% and 9% higher, respectively) than in the elbow region, but these differences were not clinically meaningful (Figure S4).

**FIGURE 5:**
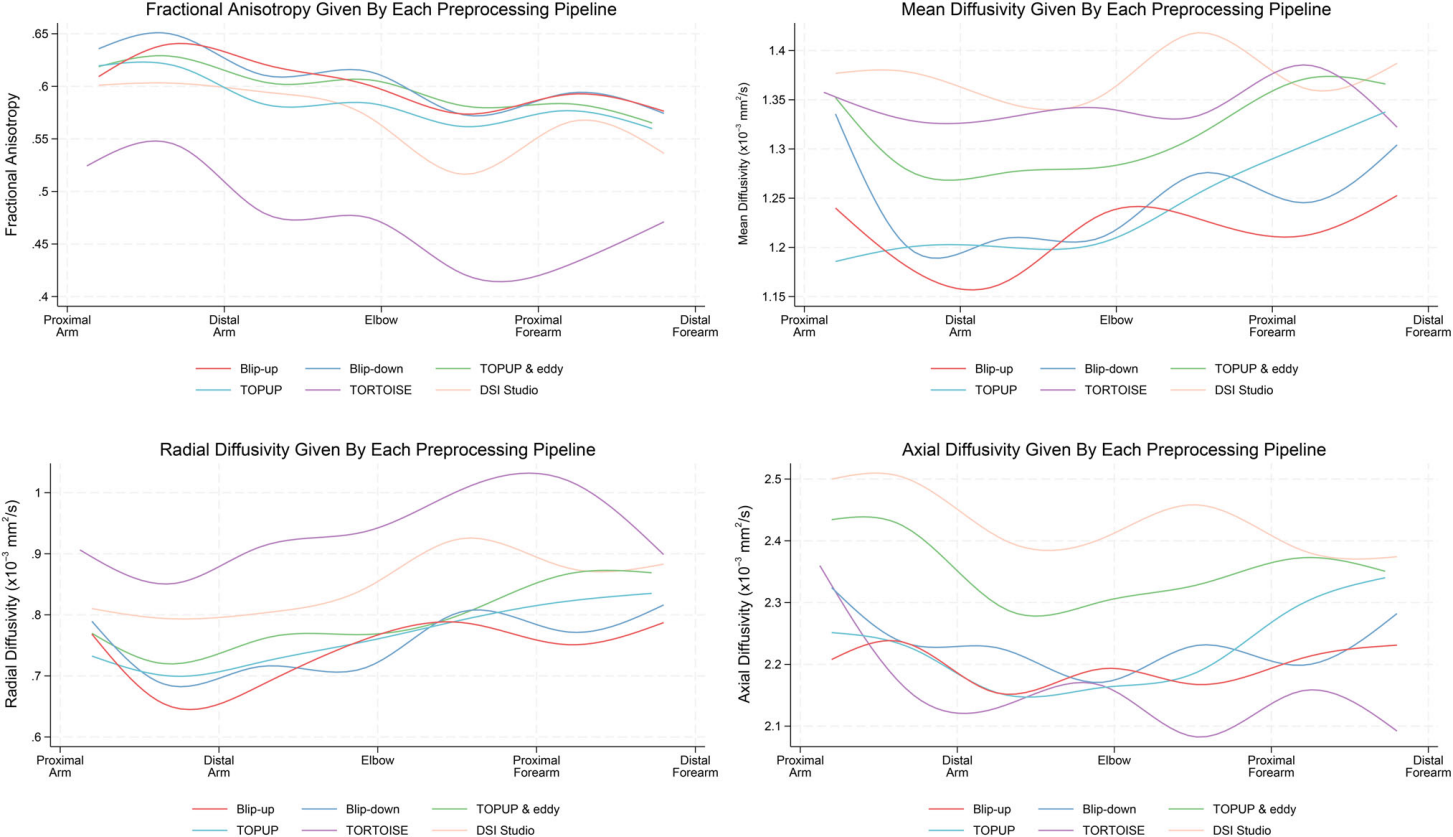
Fractional anisotropy, mean diffusivity, radial diffusivity, and axial diffusivity (plotted as cubic splines) in the peripheral nerves of the upper limb, according to their location in the limb and what (if any) pipeline was used for preprocessing.

As shown in Table S1 and Figure 5, DTI metrics from the median, radial, and ulnar nerves differed substantially according to the preprocessing pipeline used. In comparison to TOPUP & eddy, DSI Studio and TORTOISE produced estimates of the FA which were 2% (CI 2–3) and 6% (CI 5–7) lower, respectively (Figure 5). There was no differences in FA between TOPUP & eddy and TOPUP alone. The agreement in FA between pipelines was generally poor (Figures S5–S10, Pearson's *r* < 0.6; ICC <0.2). Compared to TOPUP & eddy, TOPUP generated lower estimates of MD (0.075 × 10^−3^ mm^2^/s [CI 0.053 × 10^−3^–0.096 × 10^−3^]), whereas DSI Studio yielded higher estimates of MD (0.032 × 10^−3^ mm^2^/s [CI 0.012 × 10^−3^–0.052 × 10^−3^]). MD was not different between TOPUP & eddy and TORTOISE (Figure 5). In comparison to TOPUP & eddy, DSI Studio and TORTOISE both produced higher RD values (0.053 × 10^−3^ mm^2^/s [CI 0.033 × 10^−3^–0.074 × 10^−3^] and 0.136 [CI 0.113 × 10^−3^–0.159 × 10^−3^]), respectively, whereas TOPUP reduced the RD by 0.041 (CI 0.018 × 10^−3^–0.064 × 10^−3^) (Figure 5). Compared to TOPUP & eddy, estimates of AD were lowered in TOPUP, DSI Studio, and TORTOISE by 0.145 × 10^−3^ mm^2^/s (CI 0.116–0.173), 0.010 × 10^−3^ mm^2^/s (CI 0.016 × 10^−3^–0.037 × 10^−3^ mm^2^/s), and 0.234 × 10^−3^ mm^2^/s (CI 0.205 × 10^−3^–0.263 × 10^−3^ mm^2^/s), respectively (Figure 5).

## 4 DISCUSSION

This study suggests that software used for preprocessing DTI improves the geometric accuracy of dMRI data from upper limb with respect to the anatomical ground truth, but this comes at the expense of introducing large differences in the diffusion metrics extracted from peripheral nerves. This is clinically important because the magnitude of variation in DTI parameters between preprocessing software is of the magnitude that might be attributable to disease or injury.^52^, ^53^ Consequently, in the absence of standardization of methods used by pipelines, readers should be aware of the potential for preprocessing to bias the output.

It is widely accepted that preprocessing of dMRI data derived from EPI is beneficial^17^ and improves the re‐test reliability of group studies.^54^ Furthermore, EPI artifacts are prevalent throughout the magnitude image (not just localized to the sources of classically cited distortion, e.g., air‐tissue interfaces); hence, preprocessing benefits datapoints throughout the entire volume.^55^ However, there are numerous software packages available for prepreocessing, each with different approaches and user‐specified options that can be deployable in any order or combination. This freedom means that the plausible combinations is substantial and arguably at least 15 factorial.^10^ It is well known that variability in preprocessing generates differences in the alignment of dMRI datasets with anatomical images,^11^ tensor‐based metrics,^12^ and tractograms^2^ in the brain. This in turn reduces the external validity of studies.^13^, ^14^ Although recent works suggest that there are no meaningful differences in the outputs of different pipelines,^56^ controversy still exists. Consequently, the majority (55%) of the dMRI community feels that preprocessing pipelines should be standardized.^15^ Of those advocating for standardization, 80% stated that the minimum should include corrections for artifacts arising from subject motion, eddy currents, field inhomogeneities, and thereafter, *b*‐matrix rotation should be performed.^15^ Given the importance and complexity of this topic, the ISMRM Diffusion Study Group assembled a research collaborative including 232 international scientists and clinicians. The aim was to survey the community regarding typical practices, and thereafter, collaborators were invited to preprocess 13 multi‐shell spin‐echo EPI dMRI datasets from the brain, from multi‐vendor 3T systems (GE SIGNA, Siemens Connectom and Philips Achieva) from multiple sites and sessions. The outputs from this collaborative study should provide important insight into the scale of the problem^15^ and lay the foundations for how preprocessing might be standardized.^57^

Importantly, we used ZOOMit (Siemens reduced FOV product), which deploys a dynamic excitation pulse to reduce the length of the EPI train. This has two major advantages, namely shorter TEs and distortion reduction. This may explain why we observed that preprocessing only improved the Sörenson‐Dice similarity coefficient of the DTI and T1w reference by a mean 1%. It is expected that other vendors small FOV products (e.g., GE's FOCUS or Philip's iZOOM) may perform differently and moreover, non‐reduced‐FoV EPI data may have more severe distortions and therefore, preprocessing may be of greater value in correcting the geometry. We recommend that future studies that compare the performance of preprocessing pipelines in extremity dMRI include data acquired using non‐reduced FOV products and across the entire volume.

Although one pipeline (or more specifically, a given combination of corrections within a given software pipeline) may statistically outperform other pipelines (e.g., better similarity coefficients or better agreement with reference phantoms), there are other factors that users must consider when selecting a software pipeline. These might include the cost of the software, user interface, stability, compatibility with different operating systems, exploitation of hardware acceleration, developer support and integration with other popular tools. In relation to the tools compared in this study, there are some strengths and weaknesses as we see it. DSI Studio has a user‐friendly graphical interface that requires no knowledge of coding (e.g., Python, Unix, etc), the installation is easy, and the software is stable across Windows, MacOS, and various distros of Linux. In contrast, both FSL and TORTOISE warrant some knowledge of terminal‐based scripting to install and use, which may prohibit some users. Additional difficulties are presented to users of FSL on Microsoft Windows because it requires a virtual machine or the Windows Subsystem for Linux (WSL) and allied graphical software to run. Furthermore, the graphics processing unit (GPU)‐accelerated features of FSL require additional libraries and drivers, which may be challenging for users of MacOS given the recent removal of NVIDIA compatibility. It is commendable that both DSI Studio and FSL offer timely and detailed support for users via their respective forums and training courses, which have run for several years. However, user support for TORTOISE appears to have been discontinued and no training course has been advertised for several years. Beyond the empirical performance of each software, there are wider issues that prospective users should consider before committing to a particular software package.

### 4.1 Limitations

As there is no reference standard for DTI parameter estimation, we are limited to relative comparisons, whereas we would ideally compute the absolute biases/errors from the truth. Phantoms could potentially help provide reference measurements by which to evaluate the performance of the different pipelines but mimicking the microstructure and spatial context of peripheral nerves is a challenging task that requires further development. Equally, fixed post‐mortem specimens may be helpful, but they too present unique issues, for example, reduced diffusivity and T_2_, which renders clinical EPI sequences inadequate; the difficulty maintaining physiological body temperature; the absence of artifacts related to flow, movement and breathing, which would be present in in‐vivo imaging; the degradation of microstructure in the period between death and fixation; and potential tissue changes associated with choice of fixation protocol. Overall, we acknowledge that the absence of a ground truth diminishes the impact of this study. We used the November 16th 2021 release of DSI Studio^24^ for this study; readers should note that this software suite has a high version turning rate, and since our work was completed, the “Chen” version was released, which includes the option to use FSL's TOPUP^27^ and eddy^28^ regimes for correcting datasets with reversed *b* = 0 images. Moreover, this function has been optimized to exploit multiple central processing unit (CPU) cores, it now automatically detects and configures the phase‐encoding direction (which users otherwise must prescribe manually in FSL), masks/segments of the data, and deploys applytopup if eddy cannot be run. All these functions are embedded within DSI Studio, meaning that no additional downloads/installations are needed. Moreover, this is now all achievable in Windows (without the requirement for virtualization/WSL or a native installation of FSL) and Mac (with a native installation of FSL), with the full version of eddy (to concatenated full dMRI datasets of opposing phase) forecast to be incorporated into DSI Studio soon. We enabled the replacement of outlier slices in the FSL pipeline, which is the only inter‐pipeline difference of note; although only a median of one slice per volunteer was replaced (representing 0.3% of the total slices per volunteer), this may have had a minor impact on the resultant DTI metrics. We expect that our findings may be influenced by the placement of ROIs, given that metrics were extracted from one voxel overlying each nerve, per slice. We tried to guard against this by having ROIs independently checked by multiple researchers and amended by consensus. Also, we averaged data across numerous slices, which should mitigate against such noise/variability. Nonetheless, it is plausible that variation in ROI placement explains some of the observed differences. Importantly, most packages were developed for brain imaging, principally to prepare and correct voxels in a single masked brain as part of a system or network of neurons, rather than multiple structures in the body contained in the same image where the respective signals should be separate. Therefore, there is limited work on non‐brain real‐world data in this context. Although many authors have written extensively on the importance of using a rotated *b*‐matrix,^37^, ^58^ we could not find a rotated b‐matrix or transformation matrix in the outputs of DR_BUDDI and so we used the raw *b*‐vectors, which may explain some differences. Finally, the smoothness of data from each pipeline appeared to be different, whereby TORTOISE data were noticeably smoother than others (although its documentation does not describe a smoothing function), and this may translate to differences in DTI metrics.

## 5 CONCLUSIONS

Preprocessing dMRI data from the upper limb improves geometric accuracy but the choice of the pipeline appears to introduce a source of clinically important variability in the diffusion parameter estimates from peripheral nerves.

## FUNDING INFORMATION

Ryckie Wade is an Academic Clinical Lecturer funded by the National Institute for Health Research (NIHR, CL‐2021‐02‐002). This research is also supported by the NIHR Leeds Biomedical Research Centre and University of Leeds Advanced Imaging Centre, which is funded by the Medical Research Council (MR/M008991/1) with support from the British Heart Foundation (BHF‐SP/14/7/31351) and Arthritis Research UK (ARUK‐21078). The views expressed are those of the author(s) and not necessarily those of the United Kingdom's National Health Service, NIHR or Department of Health. Sven Plein is funded by a British Heart Foundation Chair (CH/16/2/32089). Timothy Griffiths was supported by the University of Leeds Medical School Alumni Footsteps Scholarship.

## CONFLICT OF INTEREST STATEMENT

The authors declare no conflicts of interest.

## Supporting information


**Figure S1.** Examples of the regions of interest drawn on the radial (yellow), median (red) and ulnar (blue) nerves.
**Figure S2.** The columns show maps from unprocessed (blip‐up and blip‐down data) and pre‐processed datasets. The rows contain maps of quantitative anisotropy (QA), fractional anisotropy (FA), mean diffusivity (MD) and the principal eigenvector (v1) with the colors red, green and blue representing diffusion in x, y and z directions.
**Figure S3.** Raincloud plot showing the Sörenson‐Dice similarity coefficient for all datasets against the T1w reference images, stratified by the anatomical region.
**Figure S4.** Raincloud plot showing the change in Sörenson‐Dice similarity coefficient scores after preprocessing (all pipelines are shown here) against the T1w reference. The horizontal line represents no (0%) change. The boxplots show the median improvement (0·014) was small (analogous to the geometric mean of ˜1% stated in the main text) and the majority of the datapoints are clustered around minimal change (IQR −2%–4%). However, there are important outliers whereby preprocessing both improved and worsened the Sörenson‐Dice similarity coefficient substantially (the worst 5% of processed slices had 0·16 lower Sörenson‐Dice similarity coefficients, whilst the best 5% improved by 0·16. Overall, distortions in the arm and forearm were better corrected that data around the elbow, although the differences are not clinically meaningful.
**Figure S5.** A scatter plot with linear fit and Bland–Altman plot showing poor agreement for FA between datasets preprocessed with TOPUP & eddy versus DSI Studio. Pearson's *r* = 0.360, ICC = 0.075.
**Figure S6.** A scatter plot with linear fit and Bland–Altman plot showing poor agreement for FA between datasets preprocessed with TOPUP & eddy versus TORTOISE. Pearson's *r* = 0.231, ICC = 0.038.
**Figure S7.** A scatter plot with linear fit and Bland–Altman plot showing poor agreement for FA between datasets preprocessed with DSI Studio versus TORTOISE. Pearson's *r* = 0.457, ICC = 0.139.
**Figure S8.** A scatter plot with linear fit and Bland–Altman plot showing poor agreement for FA between datasets preprocessed with TOPUP versus DSI Studio. Pearson's *r* = 0.536, ICC = 0.172.
**Figure S9.** A scatter plot with linear fit and Bland–Altman plot showing poor agreement for FA between datasets preprocessed with TOPUP versus TORTOISE. Pearson's *r* = 0.2919, ICC = 0.103.
**Figure S10.** A scatter plot with linear fit and Bland–Altman plot showing poor agreement for FA between datasets preprocessed with TOPUP versus TOPUP & eddy. Pearson's *r* = 0.414, ICC = 0.098.
**Table S1.** Mean DTI metrics of the median, ulnar and radial nerves for each pre‐processing pipeline, categories by anatomical location.

## Data Availability

The raw data are available open source at https://osf.io/z29m6.
