# Supplementary material for: Comparison of distortion correction preprocessing pipelines for DTI in the upper limb
Source: Magn Reson Med. 2023 Oct 13;91(2):773–83. doi: 10.1002/mrm.29881 (PMC10952179; doi:10.1002/mrm.29881)
Supplement: Supplementary file 1 — Figure S1. Examples of the regions of interest drawn on the radial (yellow), median (red) and ulnar (blue) nerves. Figure S2. The columns show maps from unprocessed (blip‐up and blip‐down data) and pre‐processed datasets. The rows contain maps of quantitative anisotropy (QA), fractional anisotropy (FA), mean diffusivity (MD) and the principal eigenvector (v1) with the colors red, green and blue representing diffusion in x, y and z directions. Figure S3. Raincloud plot showing the Sörenson‐Dice similarity coefficient for all datasets against the T1w reference images, stratified by the anatomical region. Figure S4. Raincloud plot showing the change in Sörenson‐Dice similarity coefficient scores after preprocessing (all pipelines are shown here) against the T1w reference. The horizontal line represents no (0%) change. The boxplots show the median improvement (0·014) was small (analogous to the geometric mean of ˜1% stated in the main text) and the majority of the datapoints are clustered around minimal change (IQR −2%–4%). However, there are important outliers whereby preprocessing both improved and worsened the Sörenson‐Dice similarity coefficient substantially (the worst 5% of processed slices had 0·16 lower Sörenson‐Dice similarity coefficients, whilst the best 5% improved by 0·16. Overall, distortions in the arm and forearm were better corrected that data around the elbow, although the differences are not clinically meaningful. Figure S5. A scatter plot with linear fit and Bland–Altman plot showing poor agreement for FA between datasets preprocessed with TOPUP & eddy versus DSI Studio. Pearson's r = 0.360, ICC = 0.075. Figure S6. A scatter plot with linear fit and Bland–Altman plot showing poor agreement for FA between datasets preprocessed with TOPUP & eddy versus TORTOISE. Pearson's r = 0.231, ICC = 0.038. Figure S7. A scatter plot with linear fit and Bland–Altman plot showing poor agreement for FA between datasets preprocessed with DSI Studio versus TORTOISE. Pe [file MRM-91-773-s001.pdf]

Figure S1. Examples of the regions of interest drawn on the radial (yellow), median (red) and ulnar (blue) nerves.

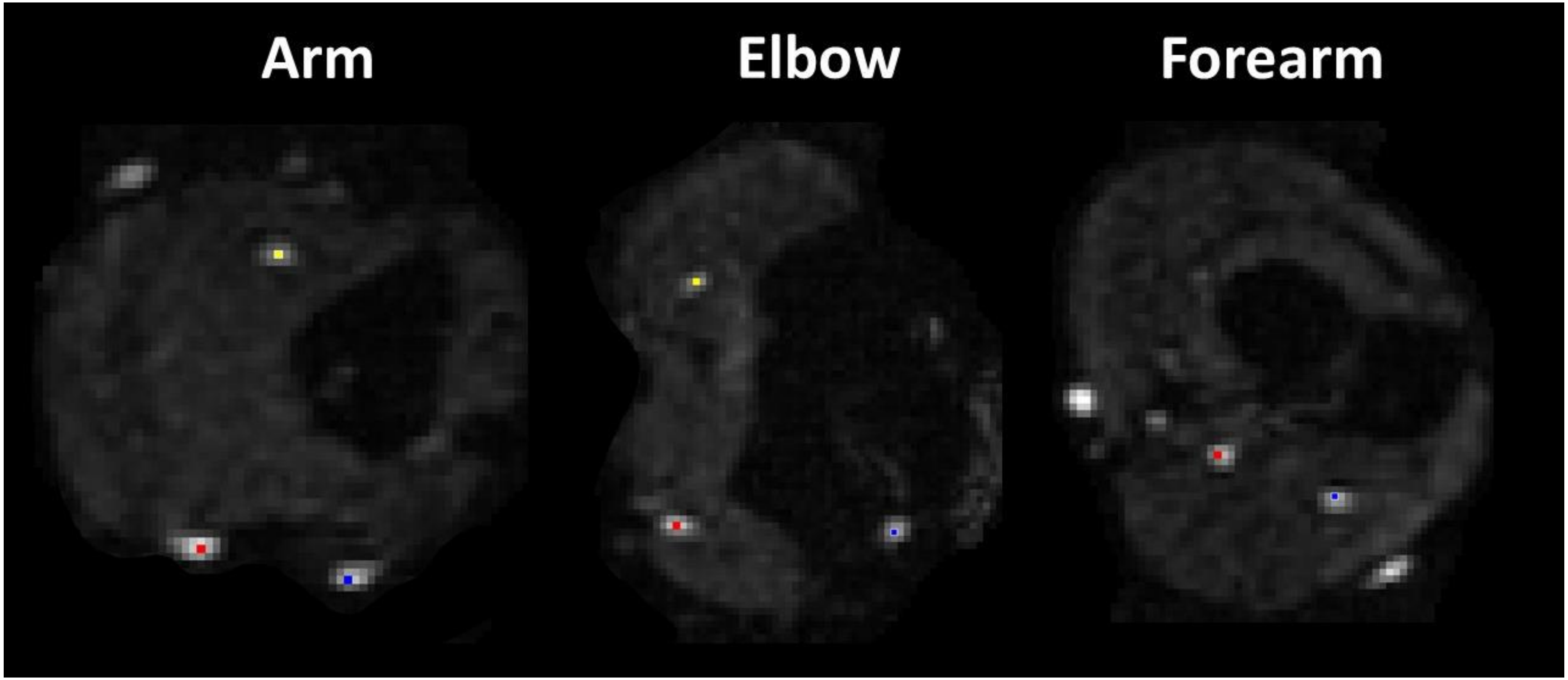

Figure S2. The columns show maps from unprocessed (blip-up and blip-down data) and pre-processed datasets. The rows contain maps of quantitative anisotropy (QA), fractional anisotropy (FA), mean diffusivity (MD) and the principal eigenvector (v1) with the colours red, green and blue representing diffusion in x, y and z directions.

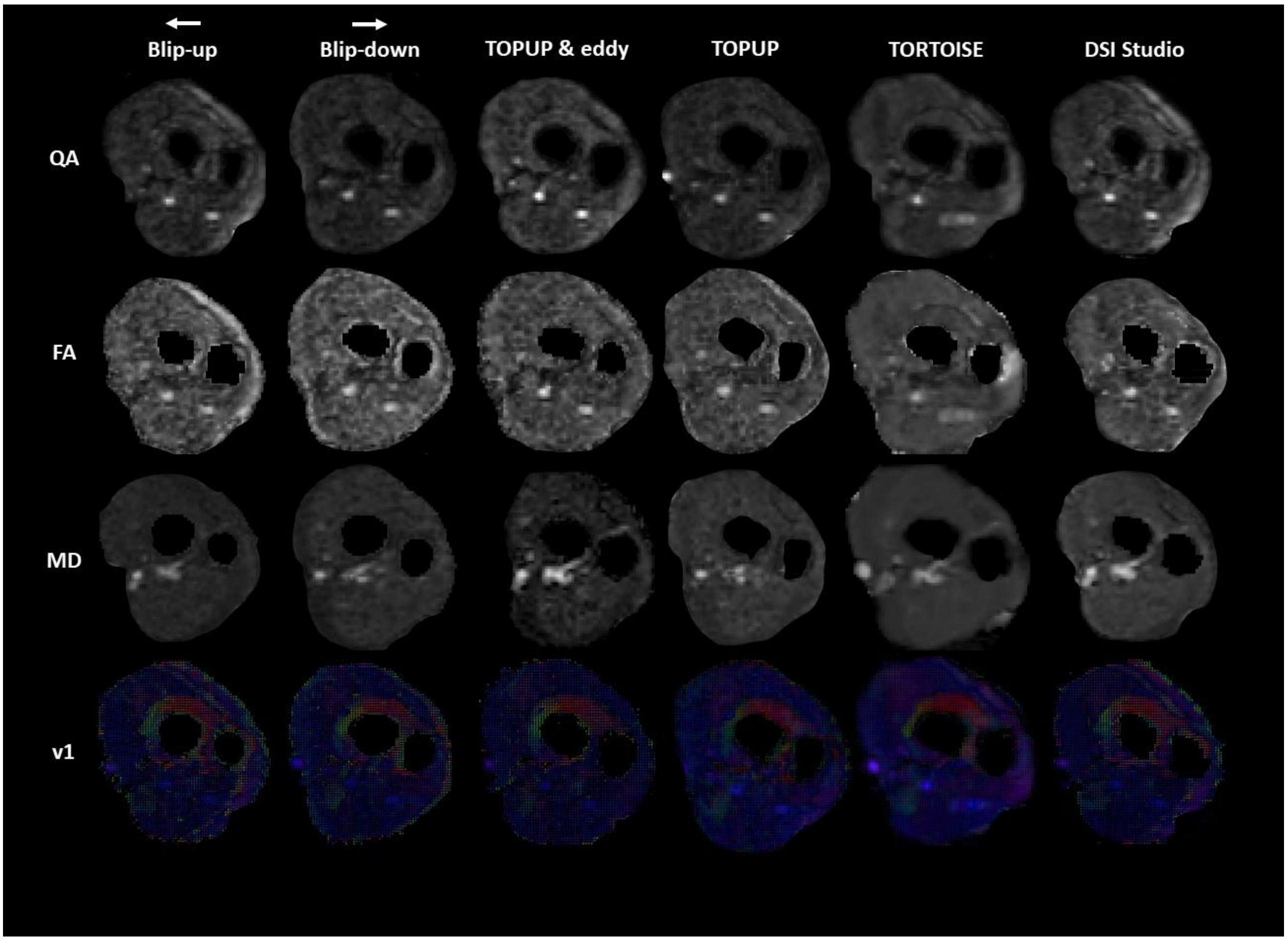

Figure S3. Raincloud plot showing the Sörenson-Dice similarity coefficient for all datasets against the T1w reference images, stratified by the anatomical region.

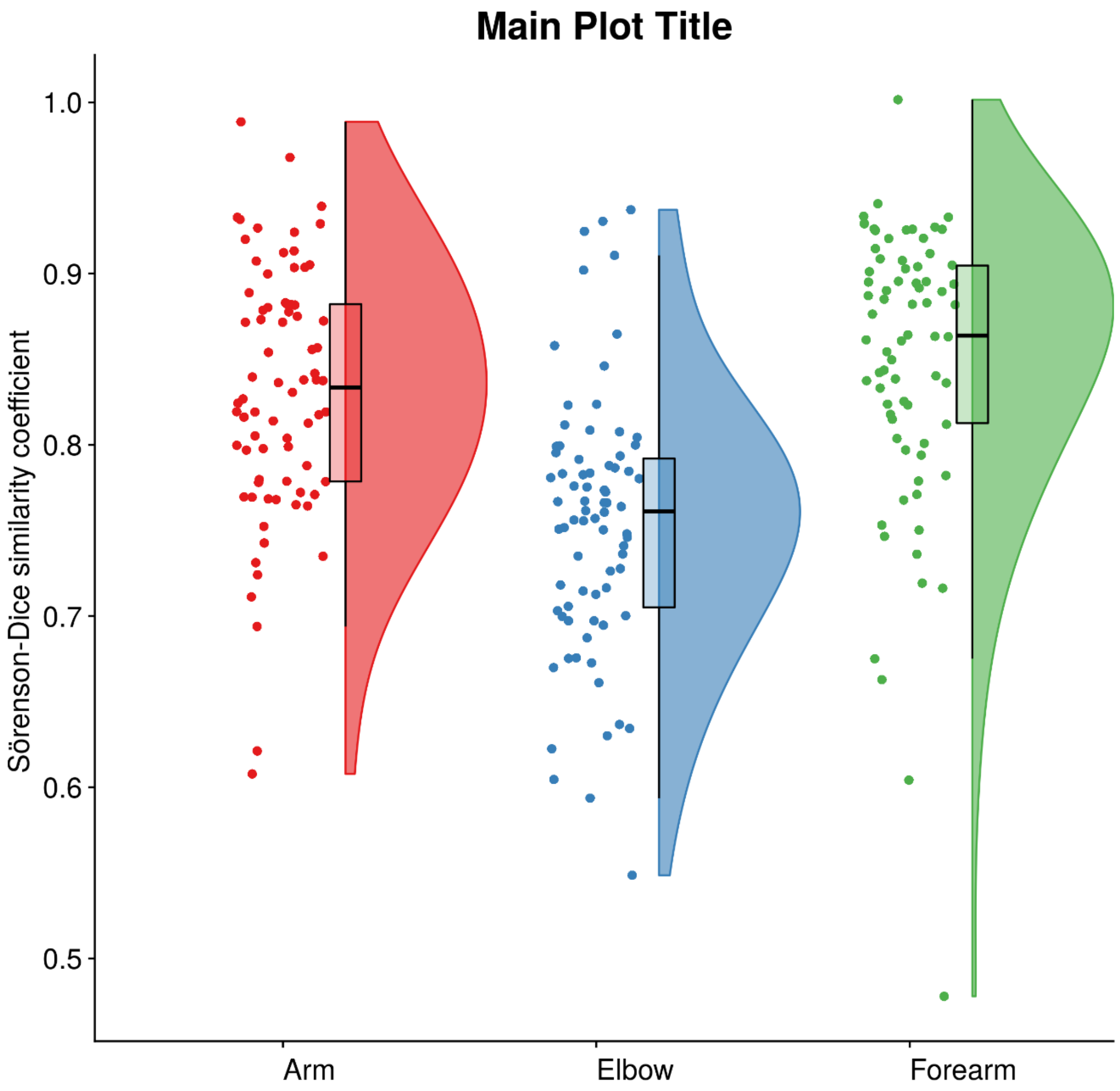

Figure S4. Raincloud plot showing the change in Sörenson-Dice similarity coefficient scores after preprocessing (all pipelines are shown here) against the T1w reference. The horizontal line represents no change. The boxplots show the median improvement (0·014) was small (analogous to the geometric mean of ~1% stated in the main text) and the majority of the datapoints are clustered around minimal change (IQR -2% to 4%). However, there are important outliers whereby preprocessing both improved and worsened the Sörenson-Dice similarity coefficient substantially (the worst 5% of processed slices had 0·16 lower Sörenson-Dice similarity coefficients, whilst the best 5% improved by 0·16. Overall, distortions in the arm and forearm were better corrected that data around the elbow, although the differences are not clinically meaningful.

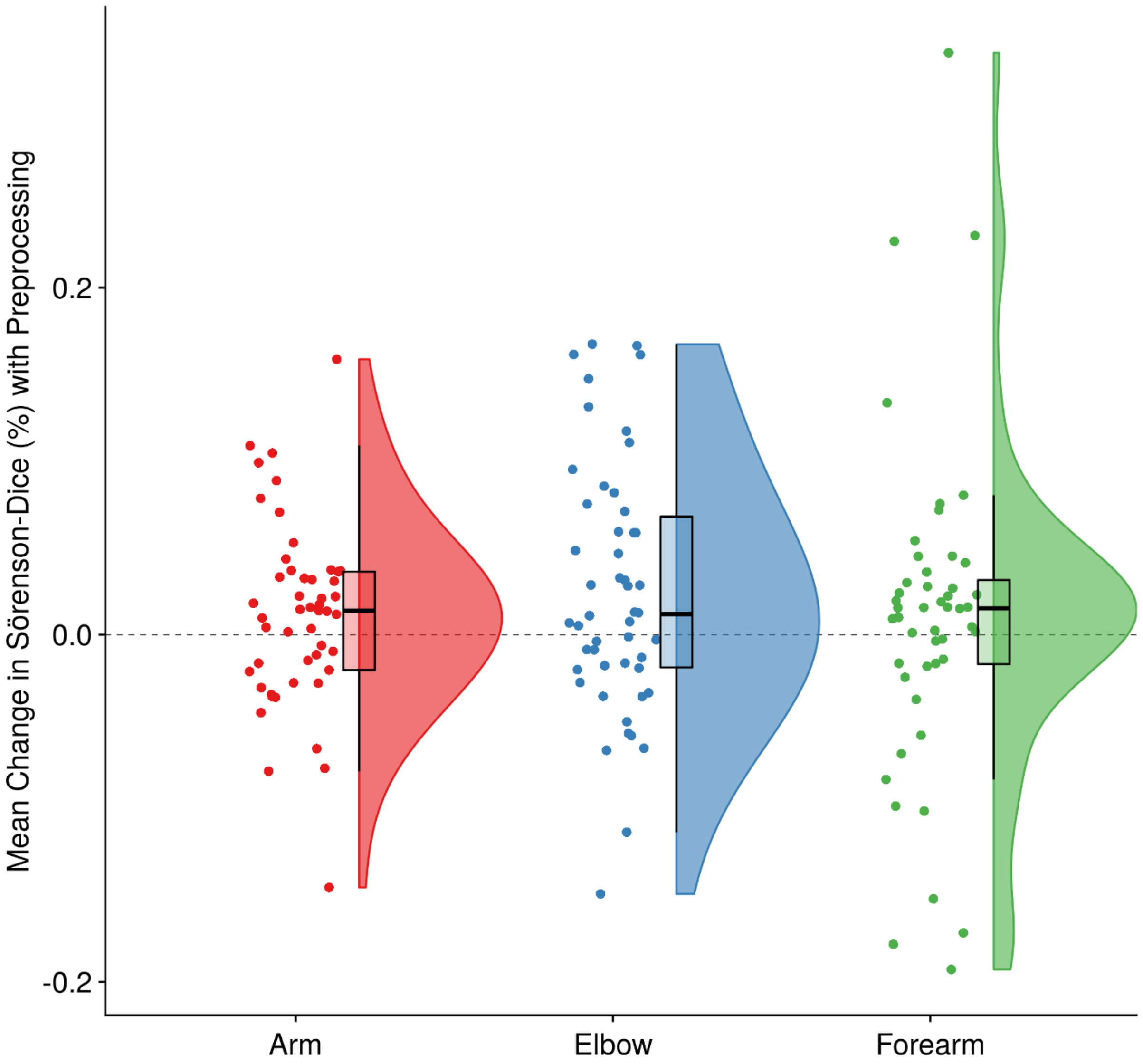

Figure S5. A scatter plot with linear fit and Bland-Altman plot showing poor agreement for FA between datasets preprocessed with TOPUP & eddy versus DSI Studio. Pearson's r=0.360, ICC=0.075.

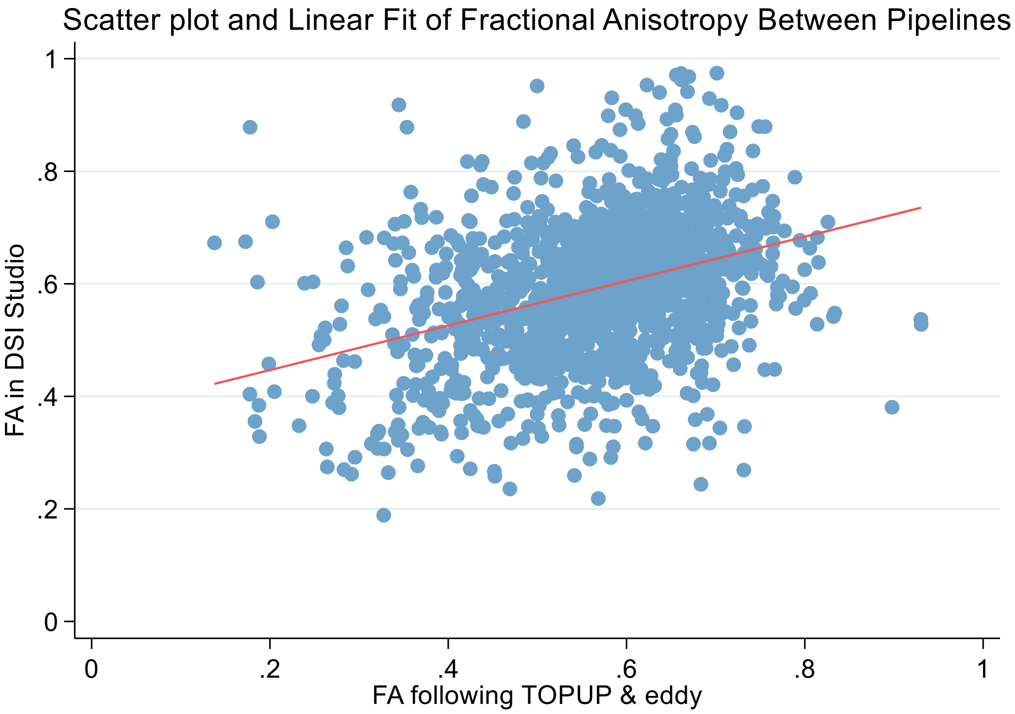

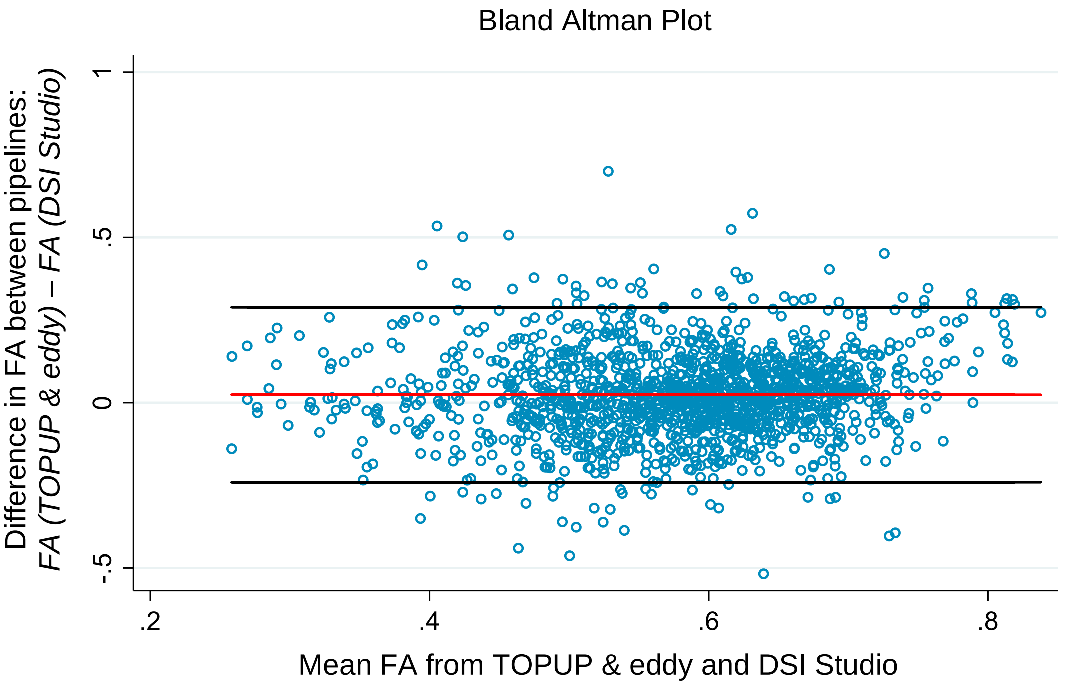

Figure S6. A scatter plot with linear fit and Bland-Altman plot showing poor agreement for FA between datasets preprocessed with TOPUP & eddy versus TORTOISE. Pearson's r=0.231, ICC=0.038.

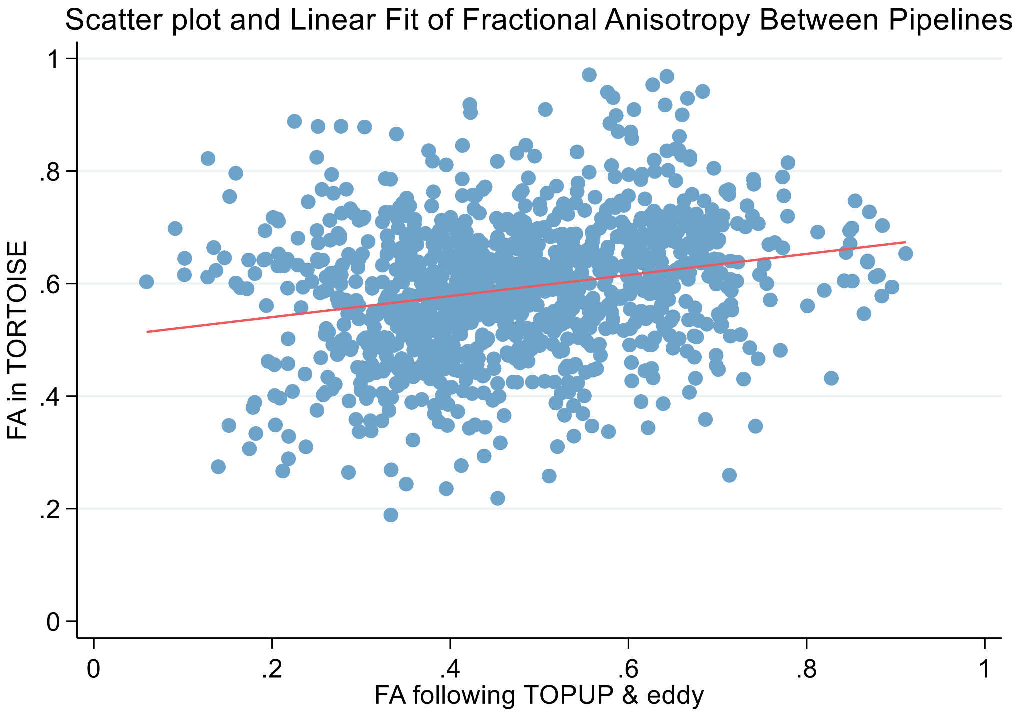

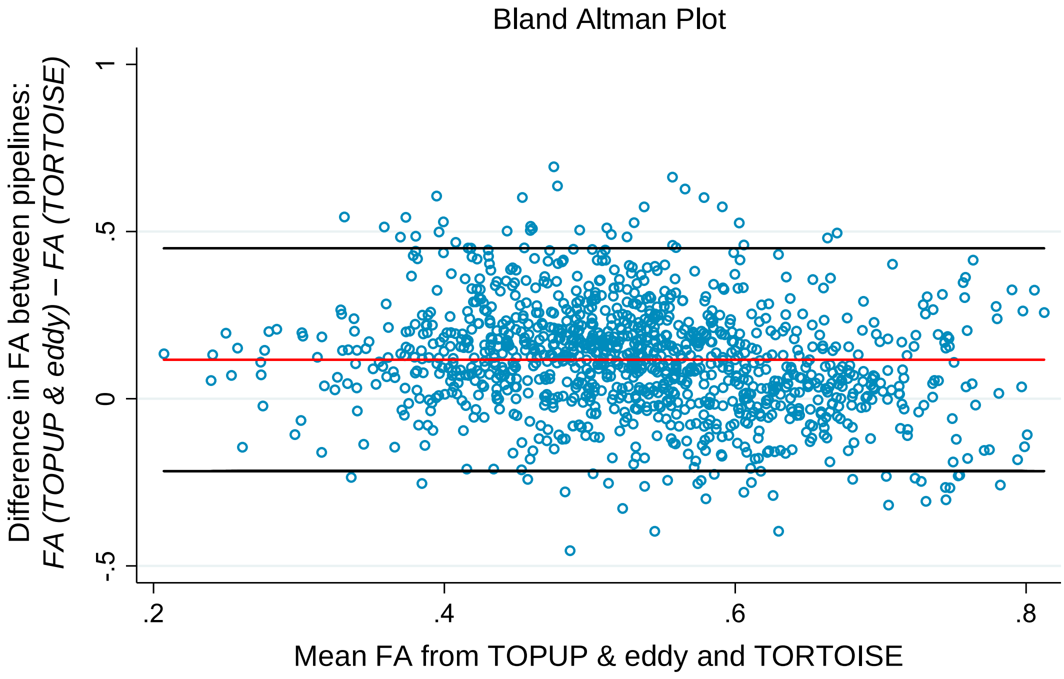

Figure S7. A scatter plot with linear fit and Bland-Altman plot showing poor agreement for FA between datasets preprocessed with DSI Studio versus TORTOISE. Pearson's r=0.457, ICC=0.139.

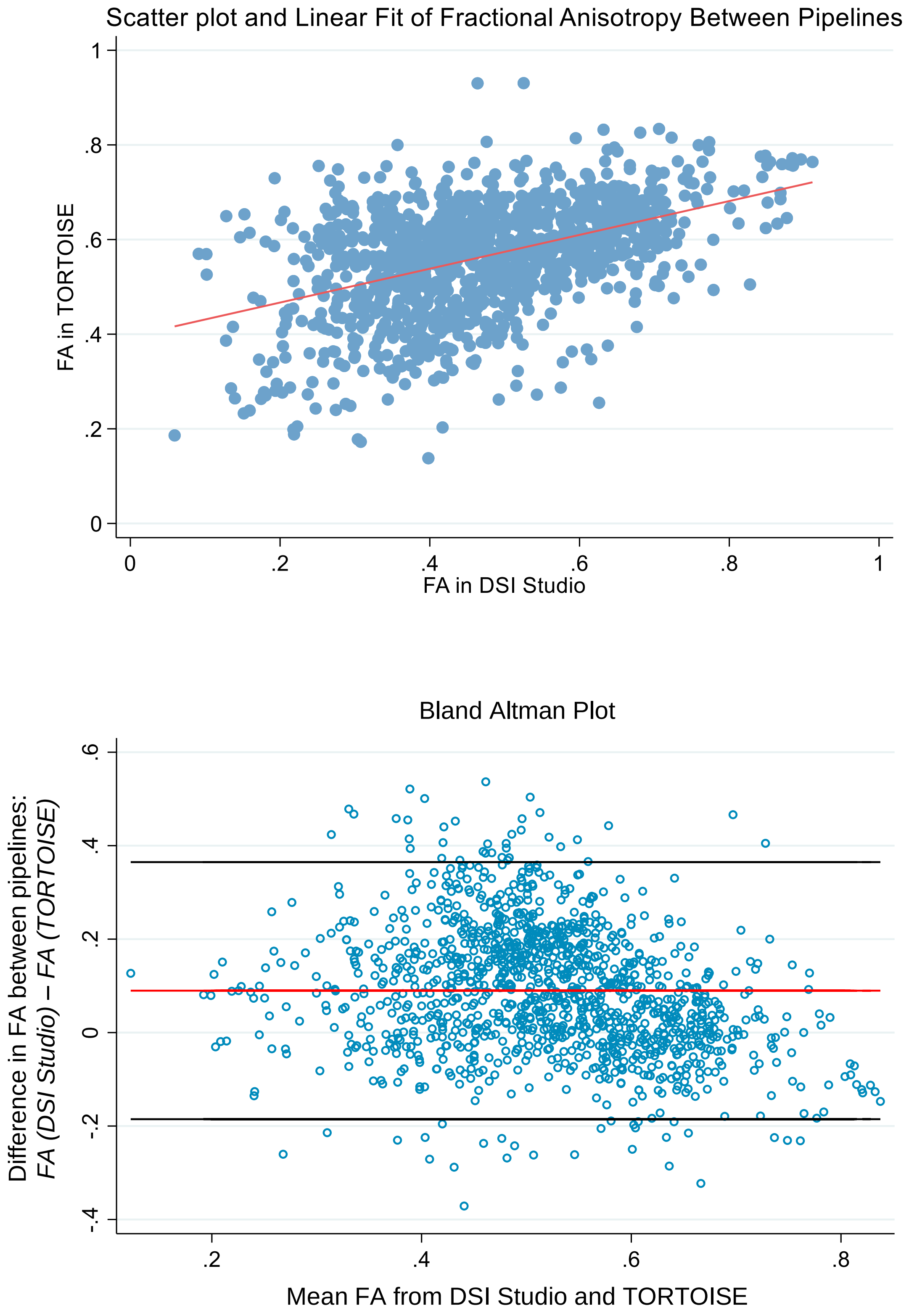

Figure S8. A scatter plot with linear fit and Bland-Altman plot showing poor agreement for FA between datasets preprocessed with TOPUP versus DSI Studio. Pearson's r=0.536, ICC=0.172.

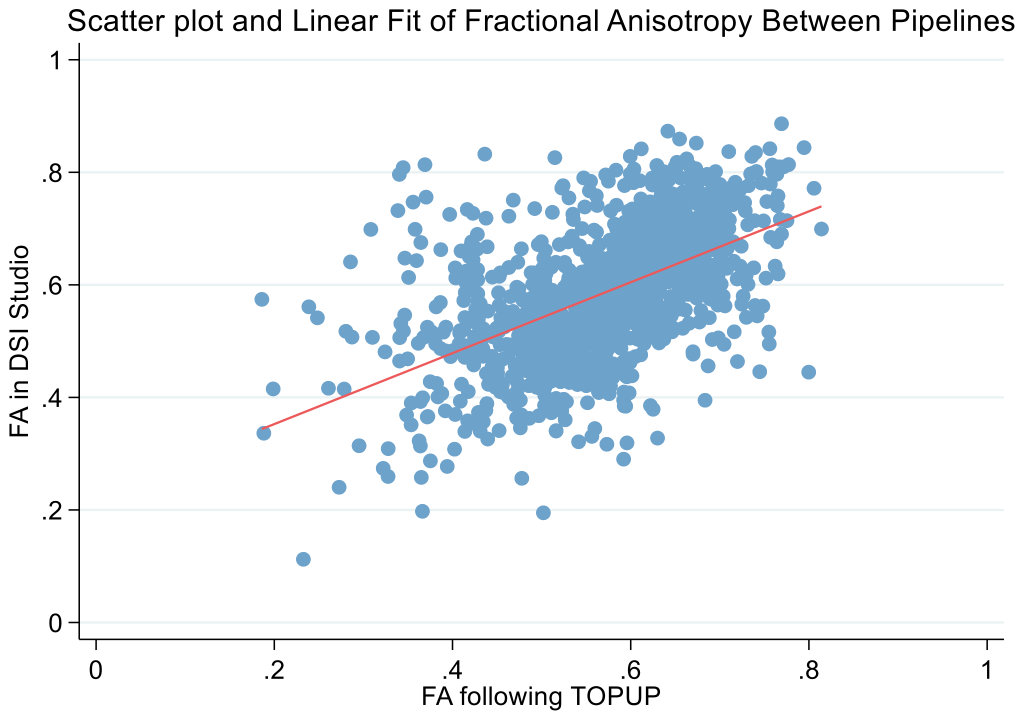

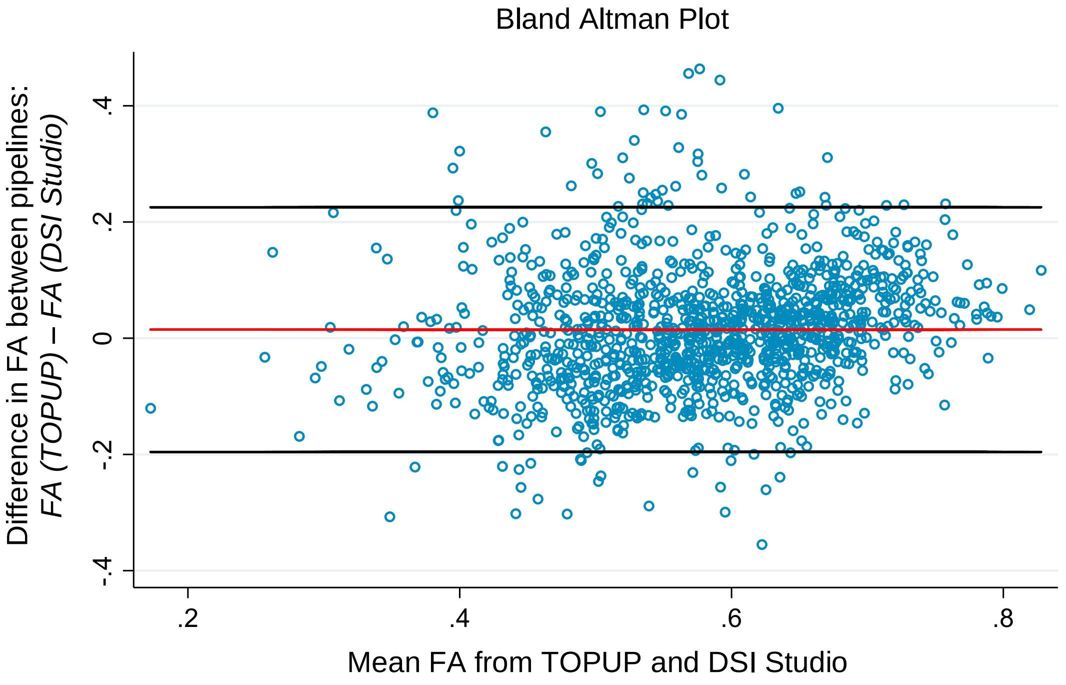

Figure S9. A scatter plot with linear fit and Bland-Altman plot showing poor agreement for FA between datasets preprocessed with TOPUP versus TORTOISE. Pearson's r=0.2919, ICC=0.103.

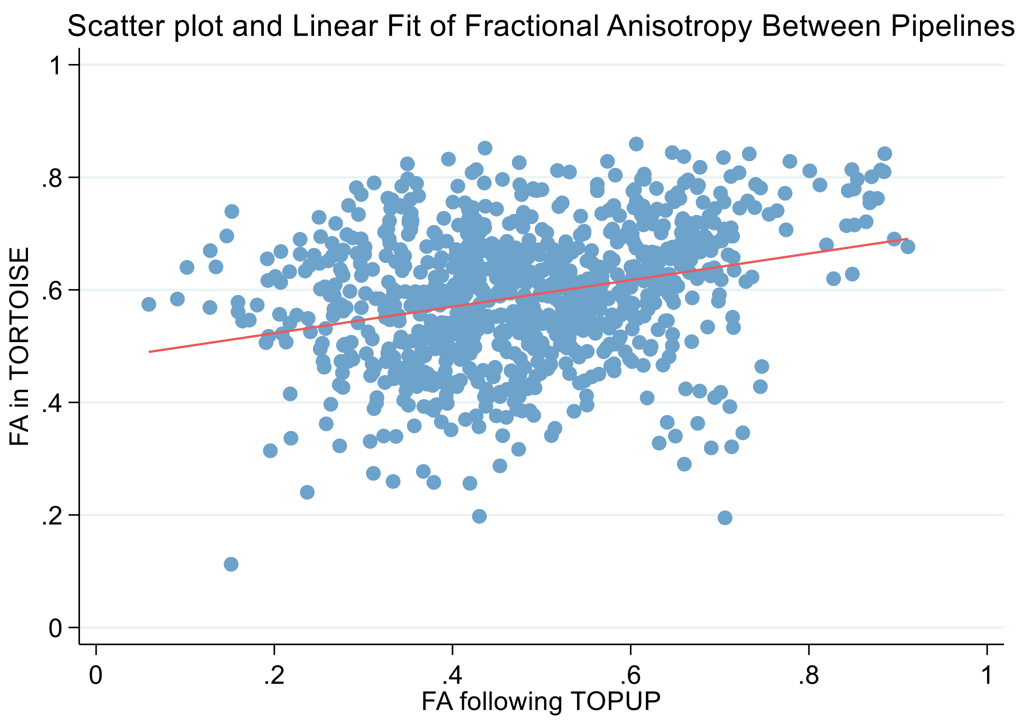

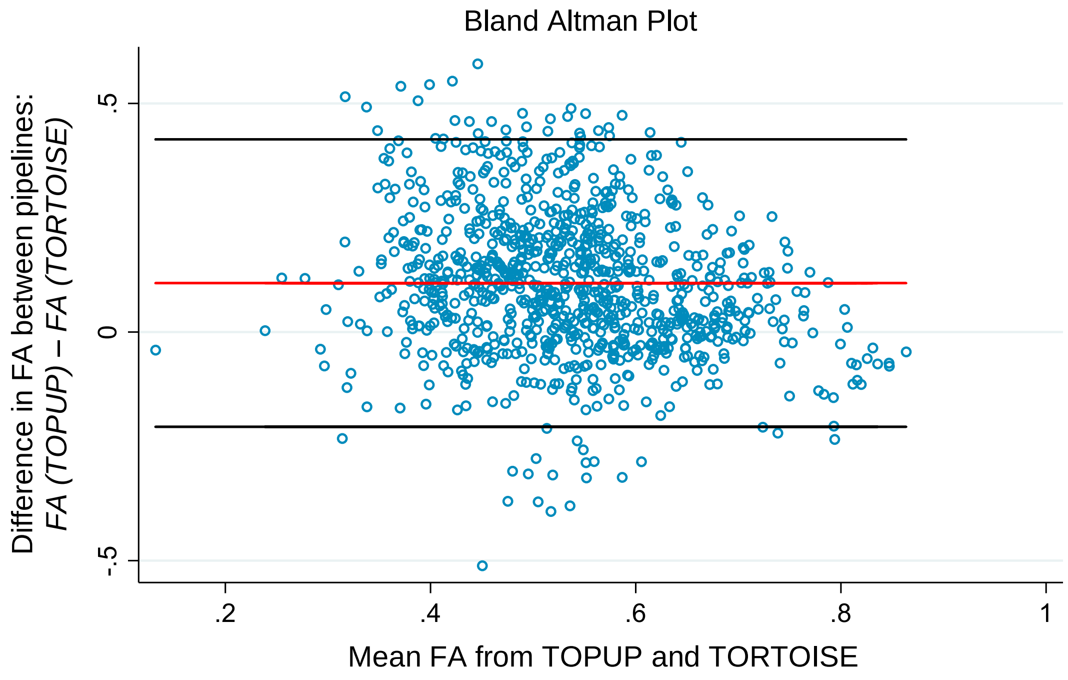

Figure S10. A scatter plot with linear fit and Bland-Altman plot showing poor agreement for FA between datasets preprocessed with TOPUP versus TOPUP & eddy. Pearson's r=0.414, ICC=0.098.

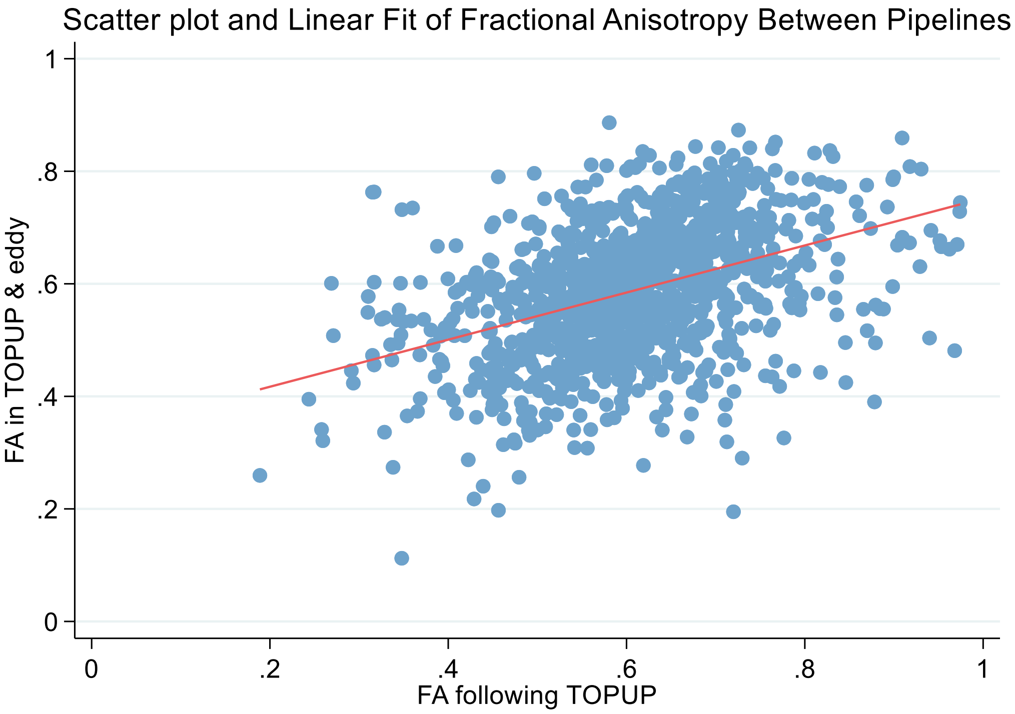

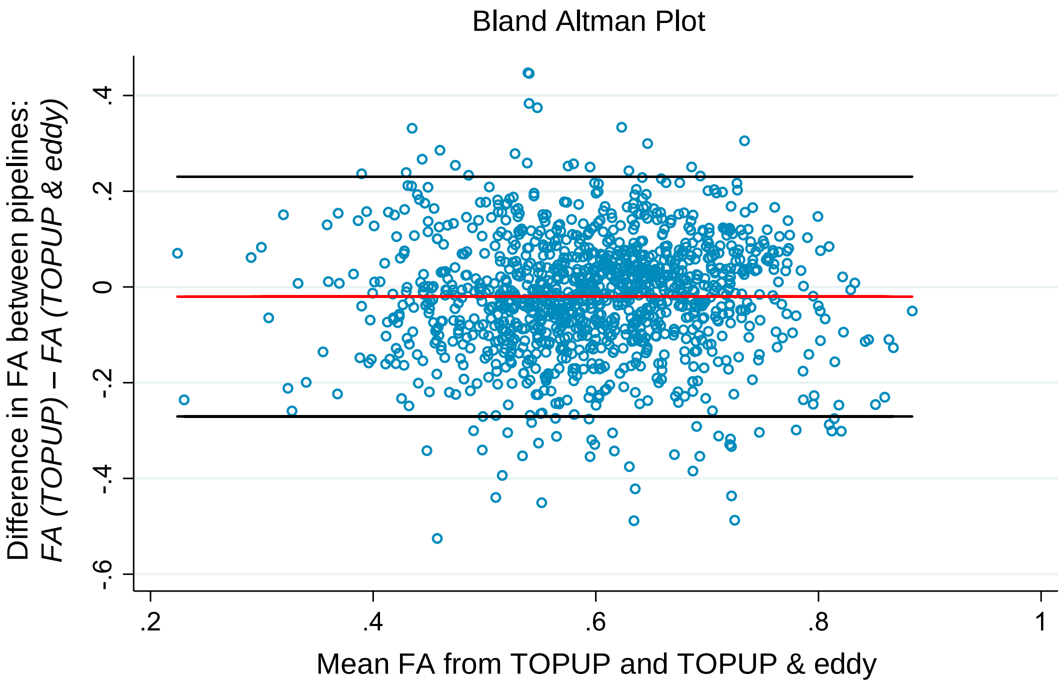

Table S1. Mean DTI metrics of the median, ulnar and radial nerves for each pre-processing pipeline, categories by anatomical location

Nerve	Location	Mean FA (SD)				Mean MD x10^{-3} mm^2/s (SD)				Mean RD x10^{-3} mm^2/s (SD)				Mean AD x10^{-3} mm^2/s (SD)			
		TOPUP & eddy	TOPUP	DSI Studio	TORTOISE	TOPUP & eddy	TOPUP	DSI Studio	TORTOISE	TOPUP & eddy	TOPUP	DSI Studio	TORTOISE	TOPUP & eddy	TOPUP	DSI Studio	TORTOISE
Median nerve	Arm	0.63 (0.15)	0.57 (0.21)	0.63 (0.07)	0.57 (0.13)	1.37 (0.46)	1.47 (0.65)	1.14 (0.25)	1.35 (0.29)	0.81 (0.48)	0.99 (0.74)	0.80 (0.17)	0.86 (0.32)	2.50 (0.53)	2.44 (0.55)	2.53 (0.49)	2.35 (0.38)
	Elbow	0·65 (0·12)	0.64 (0.16)	0·62 (0·11)	0·43 (0·17)	1·23 (0·21)	1.19 (0.47)	1·29 (0·18)	1·26 (0·33)	0.63 (0.30)	0.71 (0.53)	0.72 (0.15)	0.81 (0.39)	2.33 (0.36)	2.18 (0.46)	2.35 (0.45)	2.14 (0.47)
	Forearm	0.49 (0.10)	0.49 (0.73)	0.57 (0.11)	0.49 (0.14)	1.31 (0.23)	1.67 (0.25)	1.29 (0.21)	1.23 (0.26)	0.80 (0.21)	0.67 (0.25)	0.81 (0.16)	0.86 (0.24)	2.32 (0.37)	1.67 (0.25)	2.26 (0.49)	2.00 (0.45)
Radial nerve	Arm	0.61 (0.09)	0.62 (0.15)	0.60 (0.10)	0.46 (0.11)	1.46 (0.22)	1.24 (0.16)	1.44 (0.11)	1.38 (0.17)	0.87 (0.23)	0.76 (0.23)	0.87 (0.11)	0.99 (0.18)	2.64 (0.30)	2.27 (0.26)	2.58 (0.30)	2.16 (0.35)
	Elbow	0·49 (0·11)	0.60 (0.10)	0·45 (0·12)	0·39 (0·12)	1·41 (0·31)	1.25 (0.16)	1·56 (0·35)	1·42 (0·24)	0.90 (0.29)	0.76 (0.19)	0.99 (0.24)	1.03 (0.23)	2.36 (0.34)	2.23 (0.21)	2.45 (0.38)	2.11 (0.34)
	Forearm	0.37 (0.07)	0.49 (0.16)	0.35 (0.09)	0.32 (0.10)	1.43 (0.22)	1.26 (0.31)	1.60 (0.41)	1.49 (0.20)	1.12 (0.20)	0.89 (0.37)	1.28 (0.36)	1.21 (0.23)	2.04 (0.31)	2.01 (0.30)	2.25 (0.57)	2.05 (0.44)
Ulnar nerve	Arm	0.61 (0.11)	0.62 (0.12)	0.57 (0.07)	0.53 (0.12)	1.11 (0.27)	1.13 (0.21)	1.18 (0.23)	1.22 (0.20)	0.67 (0.24)	0.67 (0.21)	0.75 (0.15)	0.82 (0.22)	1.99 (0.40)	2.05 (0.38)	2.06 (0.46)	2.03 (0.30)
	Elbow	0·57 (0·11)	0.56 (0.12)	0·53 (0·12)	0·44 (0·12)	1·34 (0·25)	1.29 (0.31)	1·35 (0·31)	1·35 (0·26)	0.84 (0.27)	0.84 (0.30)	0.83 (0.27)	0.98 (0.30)	2.13 (0.35)	2.18 (0.42)	2.03 (0.61)	2.04 (0.46)
	Forearm	0.57 (0.08)	0.54 (0.11)	0.53 (0.12)	0.42 (0.12)	1.41 (0.18)	1.33 (0.19)	1.41 (0.22)	1.36 (0.20)	0.89 (0.17)	0.88 (0.22)	0.93 (0.19)	1.01 (0.18)	2.44 (0.30)	2.23 (0.27)	2.37 (0.45)	2.07 (0.38)
